# Missed detections of influenza A(H1)pdm09 by real-time RT–PCR assay due to haemagglutinin sequence mutation, December 2017 to March 2018, northern Viet Nam

**DOI:** 10.5365/wpsar.2018.9.3.003

**Published:** 2019-03-31

**Authors:** Phuong Hoang Vu Mai, Trang Ung Thi Hong, Hang Nguyen Le Khanh, Thuy Nguyen Thanh, Thanh Le Thi, Son Nguyen Vu, Anh Nguyen Phuong, Huong Tran Thi Thu, Cuong Vuong Duc, Mai Le Quynh

**Affiliations:** a National Institute of Hygiene and Epidemiology, Hanoi, Viet Nam.

## Abstract

**Introduction:**

There are two methods of reverse transcription polymerase chain reaction (RT–PCR) that have been the common methods to detect influenza infections: conventional and real-time RT–PCR. From December 2017 to March 2018, several missed diagnoses of influenza A(H1)pdm09 using real-time RT–PCR were reported in northern Viet Nam. This study investigated how these missed detections occurred to determine their effect on the surveillance of influenza.

**Methods:**

The haemagglutinin (HA) segments of A(H1N1)pdm09 from both real-time RT–PCR positive and negative samples were isolated and sequenced. The primer and probe sets in the HA gene were checked for mismatches, and phylogenetic analyses were performed to determine the molecular epidemiology of these viruses.

**Results:**

There were 86 positive influenza A samples; 32 were A(H1)pdm09 positive by conventional RT–PCR but were negative by real-time RT–PCR. Sequencing was conducted on 23 influenza (H1N1)pdm09 isolates that were recovered from positive samples. Eight of these were negative for A(H1)pdm09 by real-time RT–PCR. There were two different mismatches in the probe target sites of the HA gene sequences of all isolates (*n* = 23) with additional mismatches only at position 7 (template binding site) identified for all eight negative real-time RT–PCR isolates. The prime target sites had no mismatches. Phylogenetic analysis of the HA gene showed that both the positive and negative real-time RT–PCR isolates were grouped in clade 6B.1; however, the real-time RT–PCR negative viruses were located in a subgroup that referred to substitution I295V.

**Conclusion:**

Constant monitoring of genetic changes in the circulating influenza A(H1N1)pdm09 viruses is important for maintaining the sensitivity of molecular detection assays.

## Introduction

Influenza A(H1N1)pdm09 is a novel influenza detected in humans in 2009, causing the first influenza pandemic in more than 40 years. (*1*) Since then, the virus has become a seasonal influenza virus and continues to circulate worldwide in humans and pigs. (*1*, *2*) In Viet Nam, influenza A(H1N1)pdm09 spread quickly into communities in July 2009 and predominated, comprising about 85–90% of all influenza viruses during August and September of the 2009 season. After that, influenza A(H1N1)pdm02 became endemic, co-circulating with influenza A(H3N2) and B viruses. (*3*–*5*) From December 2017 to March 2018, there was circulation of both influenza A and B, with influenza A(H1N1)pdm09 again predominating in Viet Nam. Influenza A(H1N1)pdm09 was also the cause of outbreaks in other Asian countries and territories including India, Singapore, Hong Kong Special Administrative Region (SAR) and others. (*6*–*8*)

The gold standard assay for influenza diagnosis is the reverse transcription polymerase chain reaction (RT–PCR) assay. Of the two methods, real-time RT–PCR has many advantages over conventional RT–PCR. Most notably, it is time-saving, the data can be collected at the exponential phase of the reaction, and quality of amplification can be monitored during the run. Real-time RT–PCR can also detect a single target in a very small concentration of DNA or RNA because it uses a fluorescent dye that binds to targets. Despite these benefits, conventional RT–PCR is the dominant method in genetics-based diagnostic testing for influenza in Viet Nam because it is less expensive.

Sensitivity and specificity are key characteristics for diagnostic tools, and a high sensitivity is important when the test is used to identify emerging infectious diseases. Both conventional RT–PCR and real-time RT–PCR assays are rapid, sensitive methods for detecting the genetic material of influenza viruses. However, mutations in the viral genome that generate novel variants cause the sensitivity of these molecular tests to decrease and may lead to false-negative results. The A(H1)pdm09 primers and probe used in Viet Nam were adopted from the United States Centers for Disease Control and Prevention (CDC) developed in 2009 (*9*, *10*) and are commonly applied in public health laboratories throughout Viet Nam.

In Viet Nam, the national sentinel influenza surveillance system, administered by the National Institute of Hygiene and Epidemiology (NIHE) of Viet Nam’s Ministry of Health, comprises sentinel clinics linked to regional public health laboratories. Throat swabs collected from influenza-like illness (ILI) and severe acute respiratory infection (SARI) patients are tested for influenza using both conventional and real-time RT–PCR. (*4*) In this study, we investigated A(H1N1)pdm09 virus isolates that could not be subtyped using real-time RT–PCR from surveillance specimens collected in late 2017 and early 2018. These misdiagnoses may affect the sensitivity of real-time RT–PCR assays used for influenza surveillance in Viet Nam.

## Methods

### Source of samples

Between December 2017 and March 2018, there were 256 throat swabs collected from ILI and SARI patients according to WHO case definitions (*11*) as part of the national sentinel influenza surveillance system. Among 256 specimens, 60 samples were from cases of ILI and 196 were cases with SARI.

### Viral isolation

The respiratory swabs positive for influenza A(H1N1)pdm09 were inoculated on Madin-Darby Canine Kidney (MDCK) cells according to the standard operating procedures (SOPs) of the National Influenza Center (NIC) located at NIHE in Hanoi. The viruses were harvested and stored at −80 °C until analysis.

### Genetic characterization

#### Molecular testing assays

**Fig. 1:**
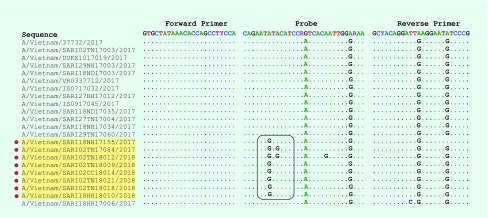
Comparison nucleotide sequences of primer–probe sets and HA sequences of influenza A(H1N1)pdm09

Ribonucleic acid (RNA) extraction was conducted on a 140µl aliquot of each sample using the QIAamp Viral RNA Mini Kit (Qiagen, Hilden, Germany) according to the manufacturer’s instructions. Influenza detection was performed using both standard molecular methods (conventional RT–PCR and real-time RT–PCR), employing specific primer and probe sets targeted to the matrix, haemagglutinin (HA) gene and nucleoprotein gene according to the SOPs of the NIC at NIHE in Hanoi (**Fig. 1**). These primers and probes were constructed following guidelines of the CDC and the World Health Organization. (*10*, *12*)

Red dots indicate identity with primer or probe sequence.

Yellow highlights indicate strains with mismatched nucleotides at position 7 (A/G) and position 9 (A/G) of probe.

#### Nucleotide sequencing and phylogenetic analysis

The influenza A(H1N1)pdm09 isolates were collected after growth in MDCK cells and their identify confirmed by using haemagglutination inhibition assay kits, provided by the CDC in 2017. The isolates with HA titre of more than 8 haemagglutinating units were selected for HA genetic analysis. RNA extraction was conducted on a 140 µl aliquot of each isolate using the viral RNA extraction kit (Qiagen, Valencia, CA, USA) according to the manufacturer’s instruction. Sanger sequencing was used to determine the nucleotide sequence of the HA gene. Briefly, cDNA was first performed using the influenza A virus universal primer (Uni 12) AGC AAA AGC AGG as described (SuperScript® III First-Strand Synthesis System, Thermo Scientific, MA, USA), followed by PCR with HA specific primer (HotStar HiFidelity Polymerase Kit, Qiagen, Valencia, CA, USA). The PCR products were purified with PCR purification kits (Qiagen, Valencia, CA, USA) and sequenced using Big Dye Terminator v3.1 (Thermo Scientific, MA, USA) on an ABI 3130 automatic DNA sequencer.

#### Analysis of nucleotide sequence data

Sequences were assembled using DNASTAR v.8.0 (*13*) and multiple sequences alignment by Bioedit v.7.0.5. (*14*) The 1140 bp haemagglutinin domain 1 (HA1) sequences were constructed into a phylogenetic tree by MEGA7, using a maximum likelihood method with bootstrap supported values. (*15*) Reference HA genes (the recent recommended vaccine strains A/California/07/2009, A/Michigan/45/2015 and others) were obtained from the National Center for Biotechnology Information in Bethesda, MD, USA. All study sequences were deposited in Genbank (MH636827 to MH636834). (*16*)

Primer and probe binding regions were aligned with recent circulating A(H1N1)pdm09 viruses to check for mismatches by using Geneious v.8.1.8 software. (*17*)

### Ethics

The routine surveillance activities were approved by the Ethic Committee and the Scientific Committee of NIHE.

## Results

### Detection of influenza A(H1)pdm09 during study period

There were 86 samples positive for influenza A by both conventional RT–PCR and real-time RT–PCR during the study period (**Table 1**). Using conventional RT–PCR for subtyping, there were 82 samples (95.4%) that were A(H1N1)pdm09 positive and four samples (4.6%) that were A(H3) positive; we were able to subtype all of these samples. Using real-time RT–PCR testing, 50 samples (58.2%) were positive for A(H1)pdm09, four samples were positive for A(H3) (4.6%) and 32 (37.2%) samples could not be subtyped (**Table 1**).

**Table 1:** Number and proportions of A(H1)pdm09-positive isolates by subtyping using real-time RT–PCR and conventional RT–PCR

-	Real-time RT–PCR Influenza A positive *n*(%)				Conventional RT–PCR Influenza A positive *n*(%)		
Sample source	Influenza A	Subtypes		Unable to be subtyped	Influenza A	Subtypes	
		H1pdm09	H3			H1pdm09	H3
**SARI patients** *n* **= 196**	**52**	**31** **(36.1)**	**3**	**18** **(21)**	**52**	**49** **(57)**	**3**
**ILI patients** *n* **= 60**	**34**	**19** **(22.1)**	**1**	**14** **(16.2)**	**34**	**33** **(38.4)**	**1**
**Total** ***n* = 256**	**86** **(100)**	**50** **(58.2)**	**4** **(4.6)**	**32** **(37.2)**	**86** **(100)**	**82** **(95.4)**	**4** **(4.6)**

ILI: influenza-like illness; SARI: severe acute respiratory infection

The distribution of subtypes by real-time RT–PCR was significantly different from that of conventional RT–PCR (χ^2^ test *p* value < 0.05).

### Phylogenetic tree of HA1 gene influenza A(H1N1)pdm09

**Fig. 2:**
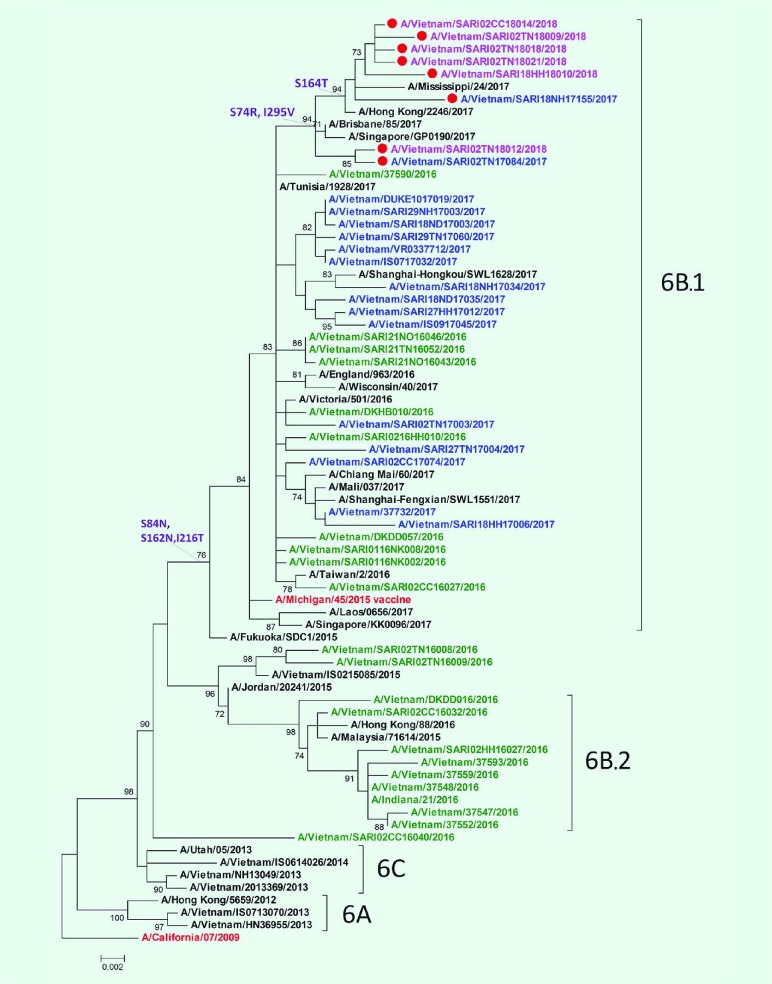
HA1 phylogenetic tree of influenza A(H1)pdm09 circulating in northern Viet Nam, 2016–2018

The HA1 phylogenetic analysis compared 23 influenza A(H1N1)pdm09 isolates, eight of which were negative for A(H1)pdm09 by real-time RT–PCR, along with other influenza A viruses that circulated in Viet Nam during 2016–2018 (**Fig. 2**). The A(H1N1)pdm09 viruses collected before this study period (September 2016 to February 2017) were mostly in the phylogenetic clade 6B, whereas most of the circulating viruses in 2017 and early 2018 were from subgroup 6B.1, represented by A/Michigan/45/2015. A small proportion of viruses were grouped in subgroup 6B.2 (**Fig. 2**).

Text in pink indicates isolates in 2018. Text in blue indicates isolates in 2017. Text in green indicates isolates in 2016. Text in red indicates vaccine strains. Red dots indicate mutant isolates.

All of the 23 influenza A(H1N1)pdm09 isolates from this study belonged to subgroup 6B.1, and amino acid analysis showed differences at residues S84N, S162N and I216T in the HA protein of subgoup 6B.1 compared to those in subgroup 6B.2 (**Fig. 2**). These isolates were closely related to viruses circulating in Australia, the Hong Kong Special Administrative Region SAR and Singapore. The eight isolates that were positive by conventional RT–PCR but negative by real-time RT–PCR gathered in new subgroups that derived from 6B.1 with a change at residue S74R and I295V. The mutation S164T appeared in only six isolates of which five had been collected in early 2018.

### Examining mismatches between primer and probe sequences used in the real-time RT–PCR assay and circulating A(H1N1)pdm09 viruses

There were no mismatches and no significant changes in the forward and reverse primers in either the negative and positive real-time RT–PCR viruses in this study. However, three mismatches at position 7 (A to G), 9 (A to G) and 17 (A to G) at the probe’s target sites were unique to the real-time RT–PCR negative viruses (**Fig. 1**); six of these had a mismatch at the 7th base, one had two mismatches at the 7th and 9th bases, and the other had three mismatches at the 7th, 9th and 21st bases (**Fig. 1**). All positive and negative influenza A(H1)pdm09 viruses by real-time RT–PCR had mismatches at the 16th (G to A) and 27th (A to G) bases in the probe’s site, implying that the real-time RT–PCR conditions likely tolerated these mismatches.

**Fig. 3:**
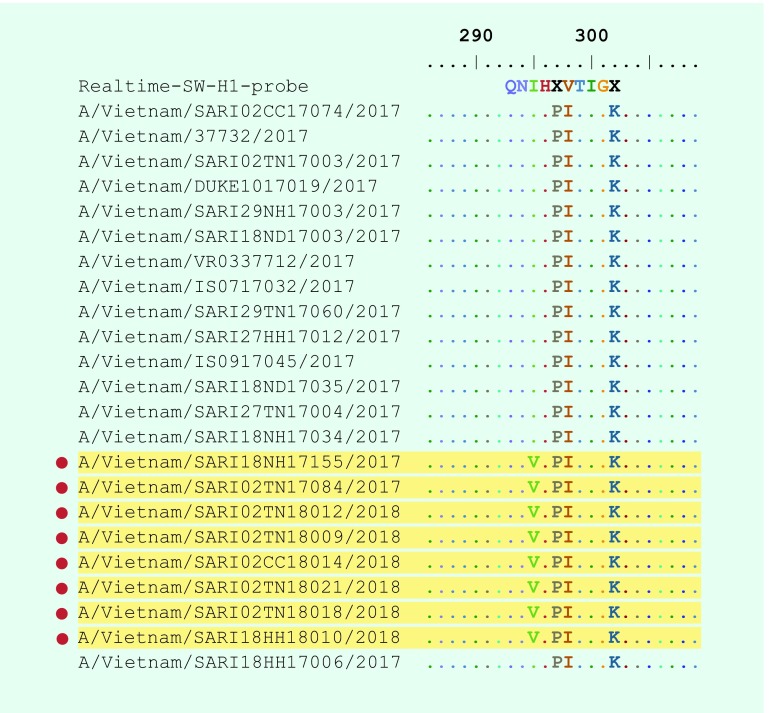
The substitutions found in the HA protein of influenza A(H1N1)pdm09 compared to the probe sequence

Analysing the HA protein of A(H1)pdm09 real-time RT–PCR negative isolates, the substitution I295V (HA1 numbering) referred to a single nucleotide mutation at position 7 in the probes (**Fig. 3**). This mutation is one of the two mutations related to new subgroups derived from group 6B.1 (**Fig. 2****, ****3**).

Red dots indicate identity with primer or probe sequence.

Yellow highlights indicate mismatched nucleotides at position 7 (A/G) and position 9 (A/G) of probe.

## Discussion

This study reports point mutations in the HA gene at specific probe sequence positions, which may have caused the false-negative subtyping results using real-time RT–PCR for influenza A(H1)pdm09 from December 2017 to March 2018 in Viet Nam. The mutation was located at the 7th base in the probe’s hybridization site that may not distinguish real-time RT–PCR negative and positive A(H1)pdm09 isolates and likely reduce the probe binding efficiency. All isolates with this mutation were in subgroup 6B.1, similar to A(H1N1)pdm09 viruses circulating worldwide recently. (*1*) Among these mutations, substitution I295V represents a mismatch between the real-time RT–PCR probes and the HA segment. However, the mutation at position 295 in HA1 protein has minor impact on RT–PCR performance; therefore, conventional RT–PCR may be the best assay to use for influenza A(H1)pdm09 detection where a new probe is being modified or developed. (*18*)

Molecular assays, such as RT–PCR, have been accepted as the gold standard diagnostic tool for the detection of influenza viruses, and real-time RT–PCR has been a key development in PCR-based technology, significantly increasing the sensitivity and reducing the turnaround time compared with conventional PCR. However, this study shows that monitoring the evolution of influenza A viruses and adapting the RT–PCR probes and primers accordingly are required to minimize the risk of missed detections. (*19*) Recently developed next-generation sequencing technology and advanced molecular assays may also be useful tools to identify virus subtypes from clinical specimens when the samples cannot be subtyped and support rapid and accurate detection of an influenza outbreak allowing for appropriate patient care and treatment. (*18*–*21*)

This study is limited in that it was based on samples collected from a small number of sentinel sites in northern Viet Nam with a small number of isolates (*n* = 23). Therefore the results may not be representative of all regions in Viet Nam or even of all areas in northern Viet Nam. However, that there was a mutation detected that may have resulted in the false-negative results by real-time RT–PCR demonstrates the influence of sequence variation in influenza viruses, which can result in probe mismatches on real-time RT–PCR assays for influenza detection in Viet Nam. This indicates a need for newly designed or modified primers and probes to effectively detect variant influenza viruses. To sustain sentinel influenza surveillance in Viet Nam, the evolution of influenza viruses should be monitored to determine genetic variants related to reduced sensitivity of diagnostic assays in a timely manner. This would be useful information not only for Viet Nam’s national surveillance system but also for other influenza surveillance systems worldwide.

The mismatch detected at position 7 on the probe nucleotide sequence site indicates that the real-time RT–PCR conditions likely tolerated these mismatches. This limits the effectiveness of real-time RT–PCR for detecting A(H1N1)pdm09 in clinical samples and isolates and should be monitored. Alternatively, conventional RT–PCR could be used as the preferred method or for those isolates where real-time RT–PCR cannot determine a subtype. It is also recommended that the primers and probes used be monitored and that a validation process for real-time RT–PCR primer–probe systems be developed.
